# MicroRNA-7 Inhibits Epithelial-to-Mesenchymal Transition and Metastasis of Breast Cancer Cells via Targeting FAK Expression

**DOI:** 10.1371/journal.pone.0041523

**Published:** 2012-08-02

**Authors:** Xiangjun Kong, Gaopeng Li, Yan Yuan, Yan He, Xiaoli Wu, Weijie Zhang, Zhengsheng Wu, Tingting Chen, Wenyong Wu, Peter E. Lobie, Tao Zhu

**Affiliations:** 1 Hefei National Laboratory for Physical Sciences at Microscale and School of Life Sciences, University of Science and Technology of China, Hefei, Anhui, P.R. China; 2 Department of Pathology, Anhui Medical University, Hefei, Anhui, P.R. China; 3 Department of General Surgery, The First Affiliated Hospital of Anhui Medical University, Hefei, Anhui, P.R. China; 4 Cancer Science Institute of Singapore and Department of Pharmacology, National University of Singapore, Singapore, Singapore; The University of Kansas Medical Center, United States of America

## Abstract

Focal adhesion kinase (FAK) is an important mediator of extracellular matrix integrin signaling, cell motility, cell proliferation and cell survival. Increased FAK expression is observed in a variety of solid human tumors and increased FAK expression and activity frequently correlate with metastatic disease and poor prognosis. Herein we identify miR-7 as a direct regulator of FAK expression. miR-7 expression is decreased in malignant versus normal breast tissue and its expression correlates inversely with metastasis in human breast cancer patients. Forced expression of miR-7 produced increased E-CADHERIN and decreased FIBRONECTIN and VIMENTIN expression in breast cancer cells. The levels of miR-7 expression was positively correlated with E-CADHERIN mRNA and negatively correlated with VIMENTIN mRNA levels in breast cancer samples. Forced expression of miR-7 in aggressive breast cancer cell lines suppressed tumor cell monolayer proliferation, anchorage independent growth, three-dimensional growth in Matrigel, migration and invasion. Conversely, inhibition of miR-7 in the HBL-100 mammary epithelial cell line promoted cell proliferation and anchorage independent growth. Rescue of FAK expression reversed miR-7 suppression of migration and invasion. miR-7 also inhibited primary breast tumor development, local invasion and metastatic colonization of breast cancer xenografts. Thus, miR-7 expression is decreased in metastatic breast cancer, correlates with the level of epithelial differentiation of the tumor and inhibits metastatic progression.

## Introduction

miRNAs are a class of evolutionarily conserved, non-coding single stranded RNAs (18–24 nucleotides) that inhibit gene expression at the post-transcriptional level [1]. Mature miRNAs operate via sequence specific interactions with the 3′ untranslated region (UTR) of cognate mRNA targets, causing degradation of mRNAs and suppression of translation [2], [3]. More than 60% of human protein coding genes have been under selective pressure to maintain pairing to miRNAs, suggesting that most mammalian mRNAs are conserved targets of miRNAs [4]. In the past decade, emerging evidences have demonstrated a central role for miRNAs in the establishment and progression of human tumors. miRNAs act as either oncogenes (e.g., miR-10b, miR-103/107 and miR-30d) [5], [6], [7] or tumor suppressors (e.g., miR-31, miR-29 and miR-200) [8], [9], [10].

Recently, miR-7 has been found to reduce EGFR (epidermal growth factor receptor) expression in glioblastoma, breast and prostate cancer cells [11], [12]. miR-7 was also observed to reduce the expression of several oncogenes including PAK1 (p21 activated kinase 1) [13] and IGF-1R (insulin-like growth factor 1 receptor) in breast cancer and tongue squamous cell carcinoma (TSCC) cell lines respectively [14]. Furthermore, miR-7 was reported to be down-regulated in glioblastoma and advanced TSCC [11], [14]. However, only a rather limited amount of clinical specimens were examined in these studies. Interestingly, Martens et al. (2008) found that miR-7 and other three miRNAs were significantly associated with aggressiveness of estrogen receptor positive (ER^+^) primary breast tumors of patients with lymph node-negative (LNN) disease [15], suggesting that miR-7 may be an oncomiR. Therefore, there is a need to further delineate the expression and function of miR-7 in breast cancer systematically.

## Results

### miR-7 is Down-regulated in Cancer Versus Normal Breast and Inversely Correlated with Metastasis

**Figure 1:**
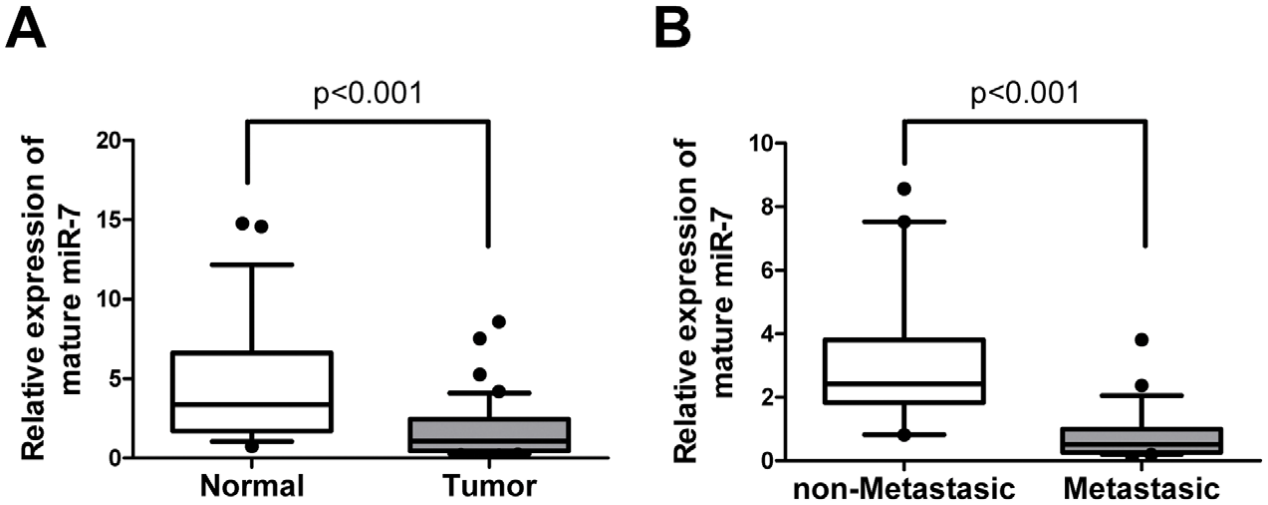
miR-7 expression is decreased in breast cancer and is associated with tumor metastasis. (A) The expression level of mature miR-7 in breast cancer (n = 42) or normal breast tissues (n = 27) was determined using quantitative PCR. Both breast cancer and normal breast are fresh tissues. Box-plot lines represent medians and interquartile ranges of the normalized threshold values; whiskers and spots indicate 10–90 percentiles and the remaining data points. (B) The relative expression of mature miR-7 in 19 non-metastatic breast cancer tissue and 23 metastatic breast cancer tissue samples. The expression level of mature miR-7 is normalized to U6 small nuclear RNA.

In an attempt to understand the role of miR-7 during breast cancer progression, we first determined the expression of miR-7 in 27 fresh specimens of normal breast tissues and 42 cases of breast cancer using quantitative reverse-transcription PCR. We observed that miR-7 expression was significantly decreased in breast cancer tissue compared with normal breast tissue (p<0.001, Fig. 1A). To determine whether miR-7 is associated with breast cancer metastasis, we further examined the miR-7 expression levels in 42 archived primary breast tumors. These tumors consisted of primary tumors resected from 23 patients with lymph node metastasis as well as tumors resected from 19 patients with no detectable lymph node metastasis. qPCR analysis revealed that patients who experienced metastasic relapse exhibited a markedly lower miR-7 expression than in those without (p<0.001, Fig. 1B). These results suggest that miR-7 may play an important role in breast cancer progression and that decreased expression of miR-7 is associated with breast cancer metastasis.

### FAK is a Direct Target of miR-7

**Figure 2:**
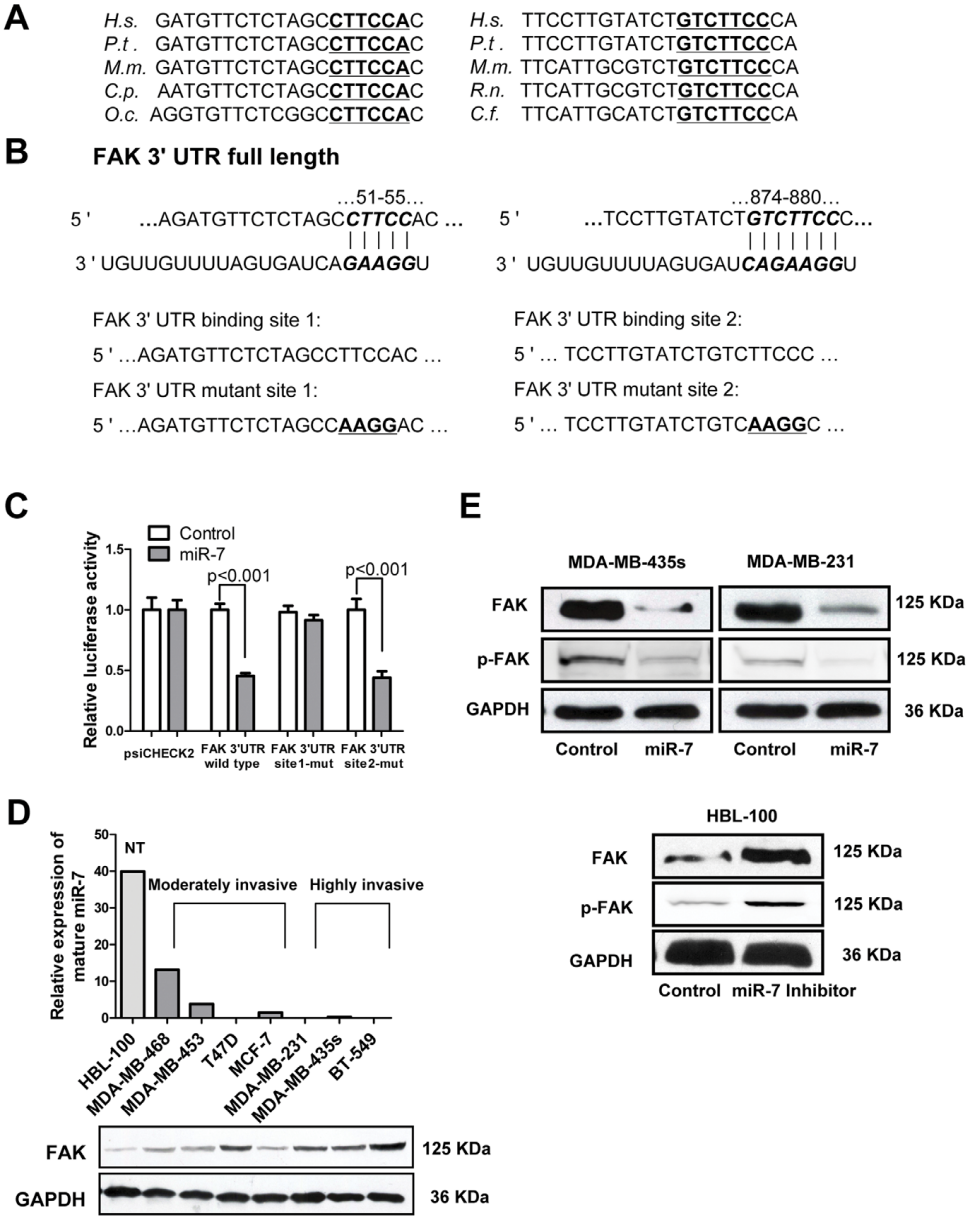
miR-7 decreases FAK expression by directly targeting its 3′-UTR. (A) The potential binding sequences for miR-7 within the FAK 3′-UTR of human (H.s), chimpanzee (P.t), mouse (M.m), guinea pig (C.p), rabbit (O.c), rat (R.n), and dog (C.f). Seed sequences are highlighted and underlined. (B) Luciferase reporter plasmids were constructed by the insertion of full length FAK 3′-UTR into the region immediately downstream of the luciferase gene. The sequences of two predicted miR-7 binding sites within the FAK 3′-UTR, including wild-type full-length UTR or mutant (highlighted and underlined) binding site are shown. (C) Relative luciferase activity was analyzed after the above reporter plasmids or control reporter plasmid were cotransfected with miR-7 mimics or control mimics in MDA-MB-435s cells. (D) Relative expression of miR-7 by quantitative PCR (top) and immunoblot for FAK expression (bottom) in the indicated cell lines. NT, non-tumorigenic. (E) Immunoblot assays of endogenous FAK protein levels in MDA-MB-435s and MDA-MB-231 cells transfected with miR-7 mimics or control mimics and those in HBL-100 cells transfected with miR-7 inhibitor or control inhibitor.

To identify downstream targets of miR-7, we performed bioinformatics analysis by use of three algorithms that predict the mRNA targets of a particular miRNA – PicTar [16], TargetScan [17], and miRanda [18]. FAK (PTK2) was one of the putative target genes that were predicted by all three algorithms (Fig. S1). FAK was of particular interest as its expression has been observed to be upregulated and associated with metastasis in several cancers including breast cancer [19]. Using RNAhybrid [20], we located two potential binding sites for miR-7 at the 3′UTR of FAK mRNA. These two sites were highly conserved in several species (Fig. 2A). In an effort to determine whether FAK is regulated by miR-7 through direct binding to its 3′UTR, a series of 3′UTR fragments including the full-length wild type 3′UTR, binding site 1 mutant and binding site 2 mutant (Fig. 2B) were constructed and inserted into the psiCHECK2 luciferase reporter plasmid. The wild type and mutant vectors were co-transfected with mature miR-7 and control miRNA in MDA-MB-435s cells. miR-7 significantly decreased the luciferase activity of wild type and binding site 2 mutant FAK 3′UTR (more than 50%) but not binding site 1 mutant FAK 3′UTR. This suggested that miR-7 interacts with the FAK mRNA 3′UTR through binding with the site 1 position because the activity of the luciferase reporter that carries a binding site 1 mutant FAK 3′UTR with substitution of four nucleotides within the miR-7 binding site was not reduced by miR-7 (Fig. 2C).

To assess the relationship between the endogenous levels of FAK and miR-7, we next determined miR-7 expression and FAK protein expression in a variety of breast cancer cell lines. FAK protein levels were low in nonmalignant human mammary epithelial HBL-100 cells and moderately invasive breast cancer MDA-MB-468, MDA-MB-453 and MCF-7 cells but relatively higher in highly invasive breast cancer MDA-MB-435s, MDA-MB-231 and BT-549 cells. In contrast, miR-7 levels were relatively high in HBL-100, MDA-MB-468, MDA-MB-453 and MCF-7 cells and much lower in MDA-MB-435s, MDA-MB-231 and BT-549 cells (Fig. 2D). The T47D cell line is a moderately invasive cell line but has a relatively low miR-7 and high FAK expression, suggestive of alternate pathways to regulate miR-7 and FAK expression in this cell line. In addition, immunoblot analyses indicated that forced expression of miR-7 significantly reduced endogenous FAK protein expression in both MDA-MB-435s and MDA-MB-231 cells (Fig. 2E, Fig. S2A and S2B). Levels of phosphorylated FAK (Tyr^397^), which is a critical event in integrin mediated FAK signaling [21] was also decreased by forced expression of miR-7 (Fig. 2E, S2A and S2B). Furthermore, after transfection of miR-7 inhibitor in HBL-100 cell, the expression of FAK and the levels of phospho-FAK (Tyr^397^) were dramatically increased (Fig. 2E and Fig. S2C). These results indicate that miR-7 expression is inversely correlated with FAK expression and activation in breast cancer cell lines and that miR-7 directly regulates FAK expression.

### miR-7 Determines Epithelial Phenotype of Breast Cancer Cells

**Figure 3:**
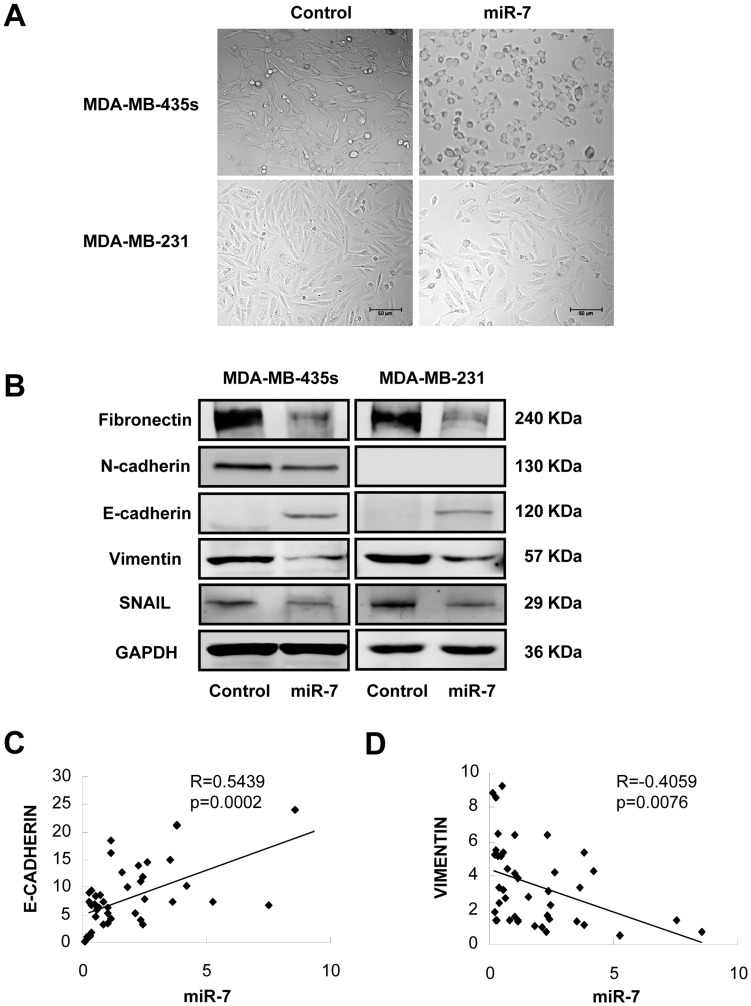
miR-7 promotes an epithelial phenotype in breast cancer cells. (A) Morphology of MDA-MB-435s and MDA-MB-231 cells transiently transfected with mature miR-7 or the control miRNA. (B) Immunoblot analysis of FIBRONECTIN, VIMENTIN, E-CADHERIN, N-CADHERIN and SNAIL in MDA-MB-435s and MDA-MB-231 cells transfected with mature miR-7 or control miRNA. (C) The correlation of mature miR-7 with E-CADHERIN mRNA in 42 breast cancer samples. (D) Correlation of mature miR-7 with VIMENTIN mRNA in 42 breast cancer samples. Pearson correlation coefficients (R) and P-values (p) are indicated.

We noticed that forced expression of miR-7 promoted striking change in the morphology of MDA-MB-435s and MDA-MB-231 cells, whereby the spindle-, fibroblast-like morphology switched to the cobblestone-like appearance of epithelial cells (Fig. 3A). The altered cell morphology produced by forced expression of miR-7 was also quantified by measuring dendricity/inverse shape factor (Fig. S2D). These morphological changes are hallmarks of reduced epithelial-to-mesenchymal transition (EMT). To determine if the molecular changes typical of a reduced EMT occurred in miR-7 expressing cells, we examined the expression of mesenchymal markers, such as FIBRONECTIN, VIMENTIN, N-CADHERIN, SNAIL and the epithelial marker E-CADHERIN in MDA-MB-435s and MDA-MB-231 cells. Immunoblot analysis showed that expression of both FIBRONECTIN, VIMENTIN and SNAIL were decreased in MDA-MB-435s and MDA-MB-231 cells with forced expression of miR-7 (Fig. 3B). The protein level of N-CADHERIN was also decreased in MDA-MB-435s cells with forced expression of miR-7. N-CADHERIN protein expression was not detectable by immunoblot analysis in MDA-MB-231 cells (Fig. 3B). Furthermore, forced expression of miR-7 increased expression of E-CADHERIN in both cell lines whereas the control transfected cells remained E-CADHERIN negative (Fig. 3B).

To determine whether the expression of E-CADHERIN correlated to miR-7 levels in breast cancer, we quantified miR-7 as well as E-CADHERIN mRNA expression in a cohort of breast cancer samples. From the cohort of 42 primary breast cancer, we observed a significant positive correlation between E-CADHERIN mRNA and miR-7 miRNA expression (Fig. 3D). We also analyzed the expression of miR-7 versus the expression of VIMENTIN mRNA in this cohort. We observed a significant inverse correlation between miR-7 miRNA and VIMENTIN mRNA expression in patient tumors (Fig. 3E). This data suggested a strong association between miR-7 expression and markers of epithelial differentiation.

### miR-7 Impairs Breast Cancer Cell Migration and Invasion *in vitro*

**Figure 4:**
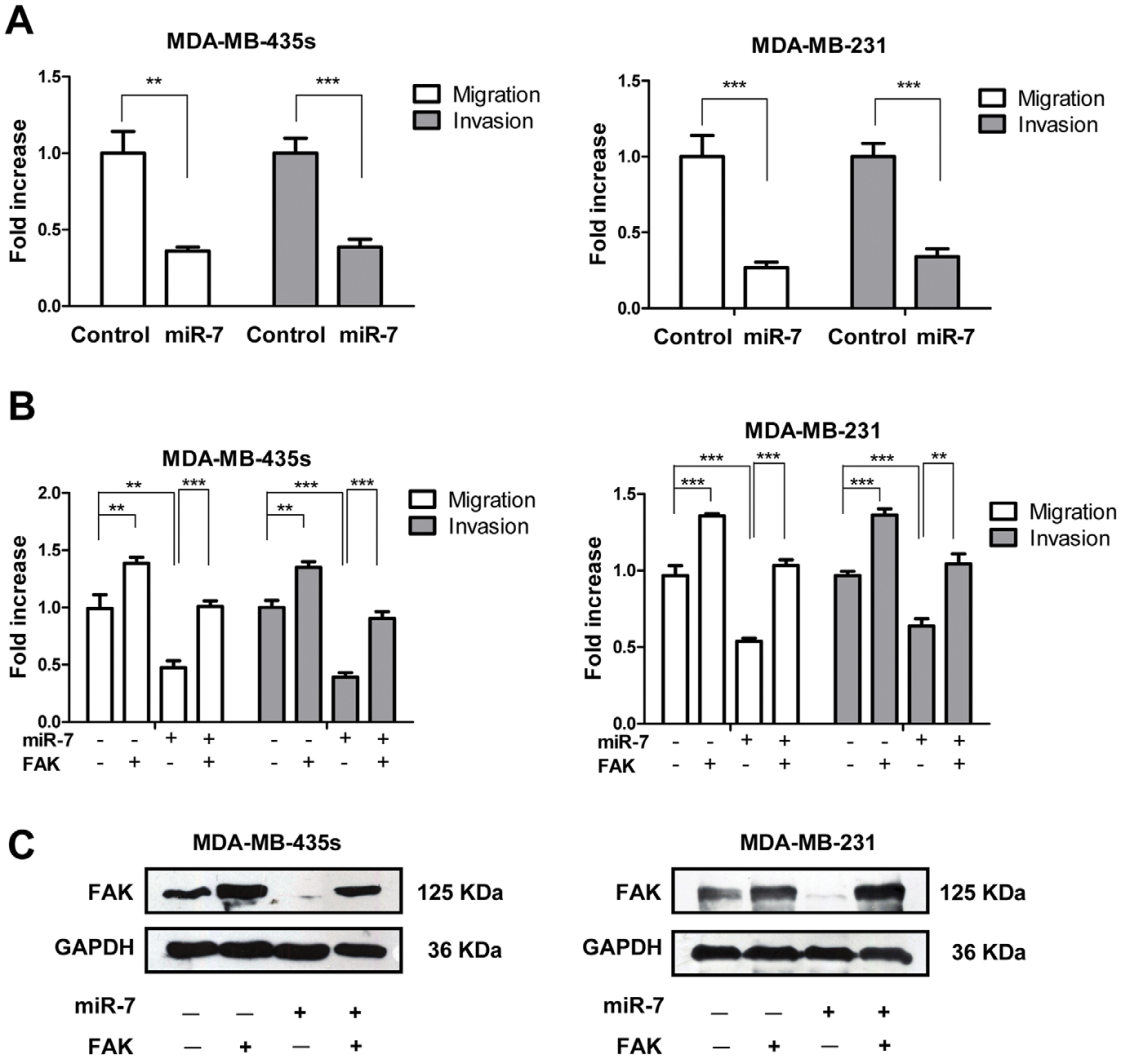
Restoration of FAK attenuates miR-7 mediated cell migration and invasion inhibition. (A) Transwell migration and invasion assays of MDA-MB-435s and MDA-MB-231 cells transfected with miR-7 mimics and control mimics. (B) Transwell migration and invasion assays of MDA-MB-435s and MDA-MB-231 cells transfected with miR-7 mimics and control mimics with or without FAK restoration. (C) Immunoblot analysis of FAK expression in MDA-MB-435s and MDA-MB-231 cells transfected with miR-7 mimics or control mimics with or without FAK restoration. ** p<0.01, *** p<0.001.

Given that the expression of miR-7 was inversely correlated with metastasis of breast cancer, we considered whether miR-7 might possess an important role in breast cancer cell migration and invasion. Transwell migration and Matrigel invasion assays demonstrated that miR-7 significantly reduced the migration and invasion capacity of MDA-MB-435s and MDA-MB-231 cells (Fig. 4A). As FAK is frequently up-regulated in breast cancer and promotes cell migration and invasion, and as miR-7 can directly regulate the expression of FAK, we next ascertained whether reduction of FAK expression might provide an explanation for the reduction of cell migration and invasion observed following forced expression of miR-7. We therefore forced the expression of miR-7 in MDA-MB-435s and MDA-MB-231 cells together with a construct containing the FAK coding sequence but lacking the 3′UTR of the FAK-encoding mRNA; and as such, this construct yielded a FAK mRNA that is resistant to miR-7. The restoration of FAK expression was confirmed through immunoblot analysis (Fig. 4C). Transwell assays indicated that restoration of FAK expression significantly abrogated miR-7 reduced cell migration and invasiveness (Fig. 4B), indicative that FAK is both a direct and functional target for miR-7.

### miR-7 Inhibits Breast Cancer Cell Growth *in vitro*

**Figure 5:**
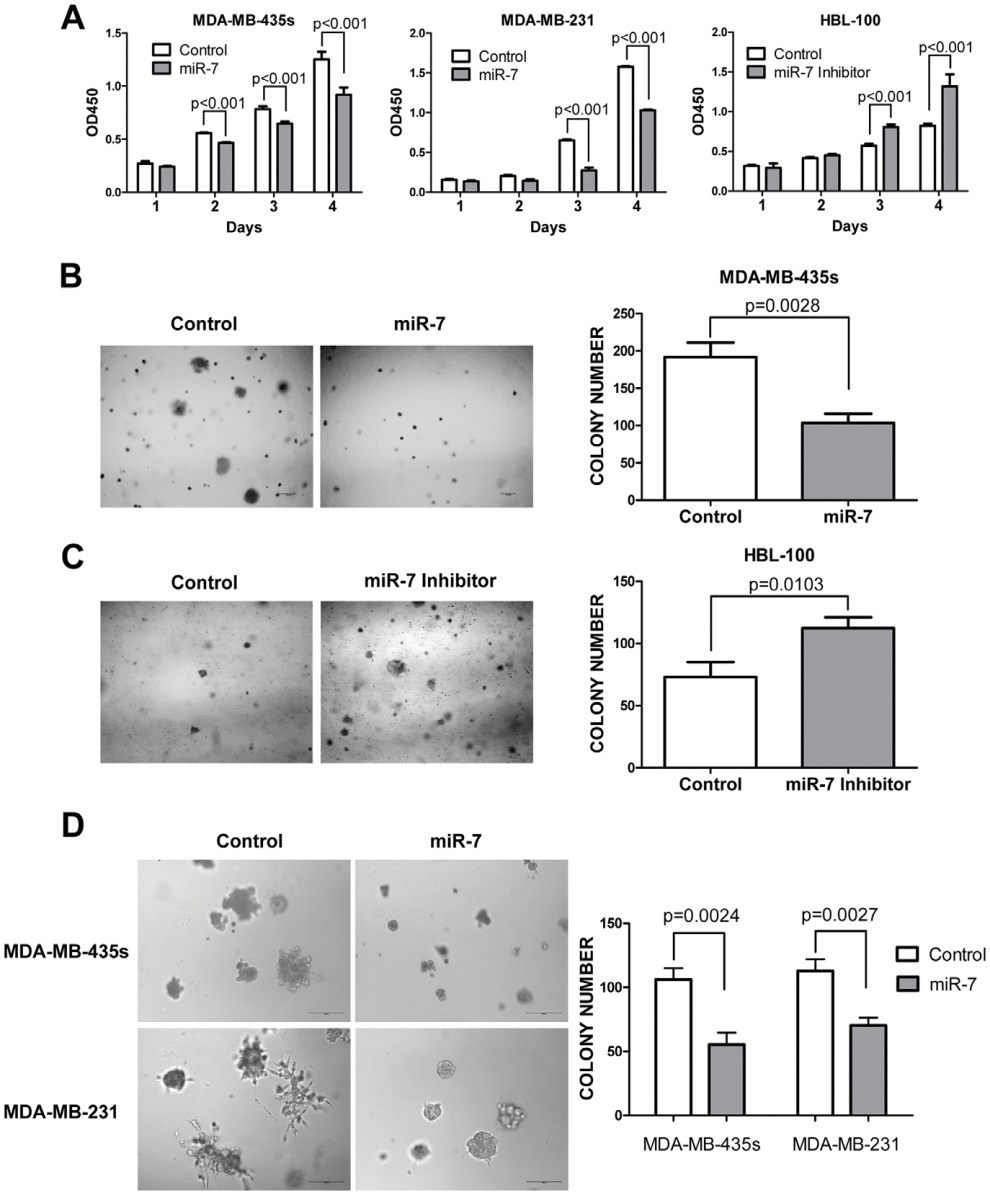
miR-7 inhibits breast cancer cell tumorigenesis *in vitro*. (A) miR-7 decreases MDA-MB-435s and MDA-MB-231 cell proliferation *in vitro* and inhibition of endogenous miR-7 expression promotes HBL-100 cell monolayer proliferation. (B) miR-7 inhibits MDA-MB-435s anchorage independent growth in soft agar. (C) Inhibition of miR-7 promotes HBL-100 cell anchorage independent growth in soft agar. (D) Morphology of MDA-MB-435s and MDA-MB-231 cells cultured in three-dimensional Matrigel after transfection with mature miR-7 or the control miRNA.

To determine the function of miR-7 in the progression of breast cancer, we sought to determine whether miR-7 may also affect the proliferation of breast cancer cells. The proliferation rates of MDA-MB-435s and MDA-MB-231 cells transfected with mature miR-7 were significantly decreased when compared with those of control miRNA transfected cells (Fig. 5A). We subsequently utilized HBL-100 cells, which possess high relative expression of miR-7 to further determine the functional effects of inhibition of miR-7. Transfection of miR-7 inhibitor increased cell proliferation in HBL-100 cells (Fig. 5A). Furthermore, transfection of miR-7 mimics in MDA-MB-435s cells produced a significant decrease in colony formation in soft agar compared with control mimics (Fig. 5B). Conversely, silencing of miR-7 in HBL-100 cells increased colony formation in soft agar (Fig. 5C). These results indicated that miR-7 inhibits breast cancer cells monolayer proliferation and anchorage independent growth *in vitro*.

We also cultured the cells in three-dimensional Matrigel [22]; growing cells in or on gels which revealed cellular behavior that are more relevant to EMT and metastasis. Less colony formation was observed for miR-7 transfected MDA-MB-435s and MDA-MB-231 cells compared to control transfected cells (Fig. 5D). Moreover, the colonies formed by control transfected cells in 3D Matrigel were larger and the cells in those colonies appeared more motile and invasive compared with colonies formed by miR-7 transfected cells (Fig. 5D). miR-7 expressing cells produced circumscribed colonies, whereas a large number of control transfected cells spread from the main bulk of the colony (Fig. 5D), suggesting that miR-7 inhibited invasive cellular behavior.

### miR-7 Decreases Tumor Growth and Suppresses Metastasis *in vivo*

**Figure 6:**
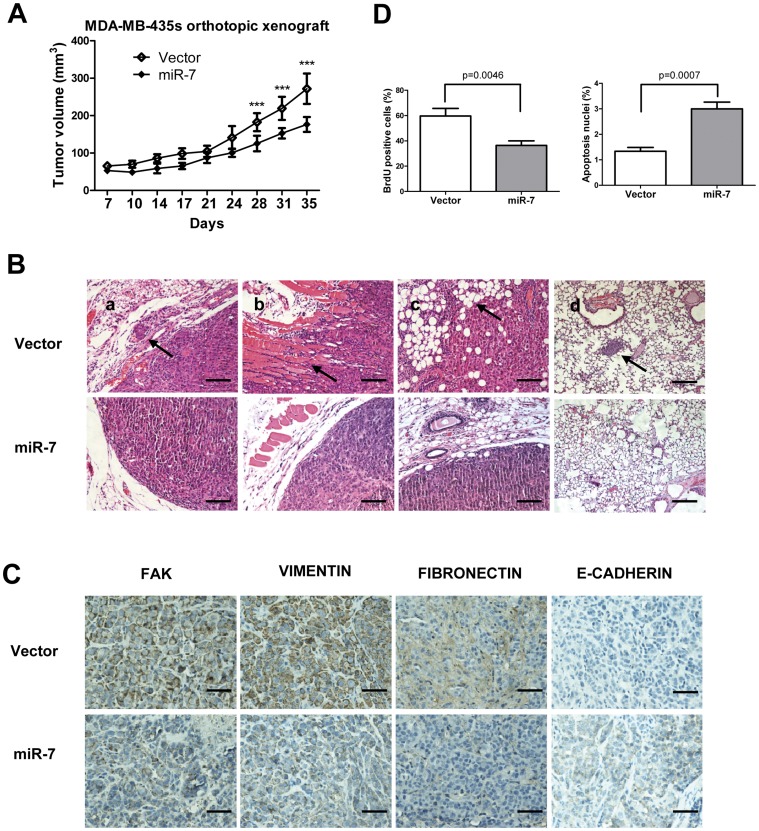
miR-7 inhibits breast cancer cell growth, local invasion *in vivo*. (A) Primary tumor growth upon orthotopic injection of 1.0×10^6^ MDA-MB-435s cells infected as indicated. n = 12 per group per time point. (B) H&E staining of primary tumors and mice lungs after orthotopic injection. Arrows: tumor cells venous (a), muscle (b) invasion and lung metastasis foci (d). (scale bar: 100 µm) (C) FAK, VIMENTIN, FIBRONECTIN, E-CADHERIN stained primary tumors after orthotopic injection. Arrow indicate metastatic foci (scale bar: 50 µm). (D) Evaluation of nuclear BrdU incorporation and TUNEL positive (apoptotic) nuclei in MDA-MB-435s tumors. ***, p<0.001.

To determine whether miR-7 regulates tumor growth and metastasis *in vivo*, we utilized xenograft models by injection of MDA-MB-435s cells, with forced expression of miR-7, orthotopically in the mammary fat pad of nude mice. Forced expression of miR-7 decreased primary tumor growth by 1.5-fold and correspondingly decreased cell proliferation as determined by immunohistochemical analysis of nuclear incorporation of BrdU (Fig. 6A and D) and increased cell apoptosis as determined by TUNEL assay (Fig. 6 D). The tumor xenografts derived from cells with forced expression of miR-7 exhibited lower expression of FAK, FIBRONECTIN and VIMENTIN than control tumors (Fig. 6C). Strikingly, a significant number of miR-7 expressing carcinoma cells exhibited staining for human E-CADHERIN (Fig. 6C). The control tumors were essentially E-CADHERIN negative (Fig. 6C). The relative expression of FAK, FIBRONECTIN and VIMENTIN between miR-7 expressing tumors and control tumors were also analyzed by quantifying DAB staining intensity (Fig. S2E). Control primary tumors displayed evidence of local invasion (Fig. 6B); however, tumors with forced expression of miR-7 were well encapsulated and non-invasive (Fig. 6B). Not only did cells with forced expression of miR-7 generate smaller primary tumors, but miR-7 also strikingly impaired in their capacity to seed lung metastasis. Cells with forced expression of miR-7 produced no metastatic lesions in contrast to control cells that formed lesions in the lungs in 2 of 6 mice with tumors orthotopically implanted (Fig. 6B).

**Figure 7:**
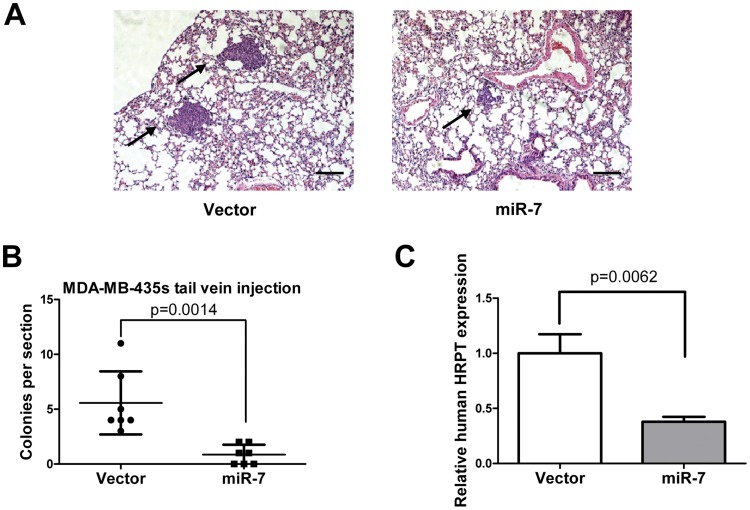
miR-7 inhibits breast cancer cell metastasis to mice lungs. (A) H&E stained sections of lungs isolated from mice that received tail vein injection of miR-7 or control infected MDA-MB-435s cells (scale bar: 100 µm). (B) Numbers of lung micrometastases per section in individual mice (each data point represents a different mouse; n = 7 mice per group). (C) Expression of human HPRT mRNA relative to mouse 18S rRNA in the lungs of the tumor-bearing mice.

We also determined the effect of miR-7 on metastasis was also attributable to effects on later steps of the invasion-metastasis cascade, independent of miR-7 influence on cellular invasion. Thus, we injected MDA-MB-435s cells with forced expression of miR-7 into the venous circulation of mice. After 30 days, MDA-MB-435s cells with forced expression of miR-7 generated fewer lung metastases than did control (Fig. 7A and B). Quantification of human HPRT mRNA also demonstrated that miR-7 decreased the number of metastatic cancer cells in the mouse lungs (Fig. 7C).

## Discussion

MicroRNAs are increasingly implicated in regulating the malignant progression of cancer by directly targeting oncogenes and tumor suppressor genes. Each miRNA can potentially interact with several mRNA targets via perfect or imperfect base pairing, primarily in the 3′-UTR portion. A number of target prediction algorithms, including TargetScan, PicTar and miRanda, relying on seed sequence pairing rules and conservational analysis, have been developed to score possible recognition sites and identify putative gene targets. However, these predictions usually yield a large number of false-positive candidates and experimental validation is thus strictly required [23]. In the present study, we located two potential binding sites (site 1 and site 2) of miR-7 in the FAK 3′UTR with RNAhybrid (Fig. 2A). However, only binding site 2 was predicted by three algorithms (PicTar, TargetScan and miRanda). Luciferase reporter assays with mutant binding site 1 and binding site 2 of FAK 3′UTR revealed that miR-7 interacts with FAK 3′UTR was through binding with site 1 (Fig. 2C), thus confirming the importance of experimental validation.

It is likely that some changes in cell adhesion occur at focal adhesion plaques which are cell/extracellular matrix (ECM) contact points containing integrin receptors, cytoskeletal components and intracellular signalling proteins such as focal adhesion kinase (p125^FAK^) [24], [25]. FAK also performs protein-protein-interaction adaptor functions at sites of cell attachment to the ECM, thereby contributing to focal-adhesion ‘scaffolding’, and transmits adhesion-dependent and growth factor-dependent signals into the cell interior [26]. In tumor cells, FAK is thought to possess a dual function, both in promoting tumor cell adhesion and in acting as a survival signal to inhibit apoptosis as the tumor develops the anchorage independent growth capacity [27]. Many studies, which are mainly based on immunohistochemical and immunoblotting analysis, have demonstrated that FAK expression is increased in cancers of the thyroid, prostate, cervix, colon, rectum, oral epithelium, ovary and breast [28]–[36]. In breast, increased expression of FAK in benign and malignant tissues is correlated with preinvasive and invasive phenotype and high FAK expression in invasive breast carcinoma is associated with an aggressive phenotype [34], [37].

The molecular mechanisms responsible for the increased FAK expression in breast cancer remain largely unknown but miR-7 may represent one mechanism. In addition to breast cancer, FAK gene amplification increased has been observed in head and neck cancer [38]. A recent report identified binding sites for the p53 tumor suppressor and NF-κB in the FAK promoter. These studies showed that p53 and NF-κB binding was able to suppress or activate expression of FAK respectively, suggesting aberrant expression or mutation of p53 and/or NF-κB could play a role in increased FAK expression in breast cancer [39]. However, we have described herein that altered posttranscriptional control of FAK mRNA by miRNAs may also contribute to increased FAK expression during breast cancer progression.

Complications from metastatic disease are the primary cause of death in breast cancer. The metastasis process depends on tumor cell intravasation, adhesion to the vessel wall, extravasation, infiltration, and proliferation into target tissue [40]. Many of these steps involve integrins, a family of transmembrane adhesion receptors composed of noncovalently linked α and β subunits [41]. Several miRNAs have reported to control cancer cell metastasis through target integrin and integrin related adhesion molecular. Valastyan et al. (2010) reported that miR-31 inhibits breast cancer metastasis via the pleiotropic suppression of a cohort of prometastatic target genes that include integrin α5 (ITGA5) [42]. Integrin β3 expression is also regulated by let-7a miRNA in malignant melanoma [43]. Integrins can alter cellular behavior through the recruitment and activation of signaling proteins such as non-receptor tyrosine kinases including FAK and c-Src that form a dual kinase complex [44]. Recently, Ding et al. (2010) demonstrated that miR-151 is frequently expressed together with its host gene FAK and can function synergistically with FAK to enhance hepatocellular carcinoma cells motility and spreading [45].

A proposed critical step in the conversion of primary tumors to metastases is attributed to the process known as epithelial-to-mesenchymal transition (EMT). EMT is a remarkable example of cellular plasticity that involves the dissolution of epithelial tight junctions, the intonation of adherens junctions, the remodeling of the cytoskeleton, and the loss of apical-basal polarity [46], [47]. Our data reveals that miR-7 inhibits EMT which promotes the conversion of highly invasive breast cancer cells with mesenchymal characteristic to the cells with epithelial properties. Transforming growth factor-β (TGF-β) has emerged as a key regulator of EMT in late-stage carcinomas, where it promotes invasion and metastasis [48]. Cicchini et al. (2007) have demostrated that FAK is required for the TGF-β induced EMT in hepatocytes [49]. Thus, FAK is an important regulatory element in the process of EMT. miR-7 has also been reported to target PAK1 in breast cancer cells [13]. PAK1 phosphorylates and modulates the subcellular localization of snail in BC cells and subsequent EMT [50]. miR-7 may therefore co-ordinately regulate breast cancer cells EMT.

Analysis of miR-7 as well as E-CADHERIN and VIMENTIN mRNA levels in breast cancer tissues reveals that miR-7 expression is an indicator of epithelial differentiation in breast cancer. Recent studies have also identified the miR-200 family as a powerful marker and determining factor of the epithelial phenotype of cancer cells by targeting the E-CADHERIN repressors ZEB1 and ZEB2 [10], [51].

While we were preparing the manuscript, it was reported that miR-7 regulated cancer cell invasion by targeting FAK expression in glioblastoma [52]. In that report, miR-7 levels in glioma tissues are inverse correlated with FAK expression when combined with work herein in breast cancer, it appears that miR-7 is a conserved miRNA that inhibits the same target gene and plays similar functions in several tumor types.

## Materials and Methods

### Tissue Samples

All patients signed informed consent approving the use of their tissues for research purposes and the study was approved by the Institutional Review Board of the Anhui Medical University. Fresh tissues including 42 breast cancer and 27 normal breast samples derived from 69 patients that underwent surgery at the First Affiliated Hospital of Anhui Medical University between 2009 and 2010 were used for the study. All tissue samples were H&E stained and had been reviewed by two independent pathologists in Anhui Medical University.

### Cell Lines

HBL-100, MCF-7, T47D, MDA-MB-468, MDA-MB-453, MDA-MB-231, MDA-MB-435s, BT-549 and HEK293T cell lines were from ATCC and cultured as recommended.

### Constructs

The human FAK cDNA expression plasmid pKH3-FAK was kind gift from Dr. Junlin Guan (University of Michigan). The ORF region of FAK cDNA was subcloned into pIRES neo3 plasmid. The 3′ untranslated region (3′-UTR) sequence of FAK was amplified from the genomic DNA of normal breast tissues and subcloned into the psiCHECK2 dual luciferase reporter plasmid (Promega). The mutant construct of FAK 3′UTR was generated using a QuikChange Site-Directed Mutagenesis Kit (Stratagene).

### RNA Isolation, miRNA and mRNA Detection

Total RNA, inclusive of the small RNA fraction, was extracted from cultured cells and clinical samples with a mirVana miRNA Isolation Kit (Ambion). Mature miR-7 was reverse-transcribed with specific RT primers, quantified with a TaqMan probe, and normalized by U6 small nuclear RNA using TaqMan miRNA assays (Applied Biosystems). mRNA expression analysis was conducted by quantitative PCR using SYBR green dye, with relative changes calculated by the ΔΔCt method.

### miRNA Gene Cloning and Ectopic Expression

The human miR-7 gene was PCR-amplified from normal genomic DNA and cloned into the pBabe-puro retroviral vector. The pBabe-amphotrophic viruses were generated by cotransfection of HEK293T cells with the pBabe constructs, pCMV-VSVG and Gag/Pol using Attractene Transfection Reagent (Qiagen). Virus was harvested at 48 and 72 h posttransfection and infections were performed in the presence of 8 µg/mL of polybrene (SigmaAldrich). Following transduction, cells were selected with 1 µg/mL puromycin (SigmaAldrich).

### Primers

Primers used were as follows: FAK ORF (GCGCGGCTAGCATGGCAGCTGCTTACCTTGACCCCA, ATAGCGGCCGCTCAGTGTGGTCTCGTCTGCCCAAGC); miR-7 ectopic expression (AGGATCCTACAGGAACACAGGACCAGA, CCGAATTCTGATAAACACGTCCATTACA), FAK 3′UTR cloning (ATCTCGAGGCCTCCCCTAGGAGCACGTCTT, GCGCGGCCGCTTTACTGGTAACACCTTTTTAAT), E-CADHERIN, VIMENTIN and GAPDH quantitative PCR: E-CADHERIN (CTGAGAACGAGGCTAACGTC, TGTCCACCATCATCATTCAATA); VIMENTIN (AGACAGGCTTTAGCGAGTTATT, GGGCTCCTAGCGGTTTAG); GAPDH (TGCACCACCAACTGCTTAGC, GGCATGGACTGTGGTCATGAG). For the *in vivo* xenograft, the following primers were used: hHPRT(TTCCTTGGTCAGGCAGTATAATCC, AGTCTGGCTTATATCCAACACTTCG); mouse 18s rRNA(GAAACGGCTACCACATCC, ACCAGACTTGCCCTCCA).

### Oligonucleotide and Plasmid Transfection

miRNA mimics (Genepharma, Shanghai, China) and miScript miRNA inhibitor (Qiagen) were transfected using HiPerFect Transfection Reagent (Qiagen) following the manufacturer’s instructions. miRNA mimics and plasmid co-transfection were performed by using Lipofectamine 2000 (Invitrogen). Twenty-four hours after transfection, cells were plated for proliferation, soft agar, migration, invasion assays or harvested for the luciferase reporter assay. Cells were harvested for RNA and protein analyses at forty-eight hours after transfection.

### *In vitro* Migration and Invasion Assay

For transwell migration assays, 10×10^4^ cells were plated in the top chamber with the non-coated membrane (24-well insert; 8 µm pore size; BD Biosciences). For invasion assays, 2×10^5^ cells were plated in the top chamber with Matrigel-coated membrane (24-well insert; 8 µm pore size; BD Biosciences). In both assays, cells were plated in medium without serum, and medium supplemented with 10% serum was used as a chemoattractant in the lower chamber. The cells were incubated for 24 to 36 hours and cells that did not migrate or invade through the pores were removed by a cotton swab. Filters were fixed with 90% ethanol, stained by 0.1% crystal violet, photographed and cells numbers were counted.

### Cell Proliferation and Soft Agar Colony Formation Assays

Cell proliferation was determined by Cell Counting Kit-8 (Dojindo, Shanghai, China) according to the manufacturer’s instructions. For soft agar colony formation assay, cells (5×10^3^) in 1.5 mL medium supplemented with 0.35% agarose were layered on a 1.5 mL base medium with 0.5% agarose. Soft agar assays were performed in six well plates and in triplicate. Cells were cultured for 14 days and colonies were counted.

### Three-dimensional Matrigel Culture

Eight hundred cells per well were plated in 10% FBS medium supplemented with 4% Matrigel (BD Bioscience) in a 96-well plate which coated with Matrigel before addition of the cells. Matrigel-containing (4%) medium was renewed every 3 d until the experiment was terminated after 10 d.

### Orthotopic Injection

The animals were maintained in a pathogen-free barrier facility at the Aninal Center of the University of Science and Technology of China (USTC), and closely monitored by animal facility staff. All animal work procedures were approved by USTC Ethics Committee for Animal Care and Use (Protocol number :USTCACUC1201040) and were performed in accordance with the regulations of animal care of USTC and conformed to the legal mandates and national guidelines for the care and maintenance of laboratory animals.

MDA-MB-435s cells (2×10^6^ cells in 100 µL PBS) were injected orthotopically into the each side mammary fat pad of female BALB/c-nu/nu mice (Slaccas, Shanghai, China). Each group consisted of six mice. Tumor growth rates were analyzed by measuring tumor length (L) and width (W), and calculating volume through the use of the formula LW^2^/2. Six hours before sacrifice, mice were i.p injected BrdU solution at the 100 µg/g body weight.

### Tail Vein Injection

MDA-MB-435s cells (1×10^6^ cells in 200 µL PBS) were injected directly into the lateral tail vein of 6- to 8-week-old female BALB/c-nu/nu mice. Each group consisted of eight mice. Mice were sacrificed on day 35 (orthotopic injection) or day 30 (tail vein injection) and mammary tumors, mice liver and lung were fixed in 10% neutral buffered formalin and embedded in paraffin for histology examination or were frozen at −80°C in RNALater (Ambion) for RNA extraction and qPCR analysis.

### Immunohistochemistry

Formalin-fixed, paraffin-embedded tissue was cut into 5 µm section, de-paraffinized in xylene, rehydrated through graded ethanol, quenched for endogenous peroxidase activity in 3% (v/v) hydrogen peroxide, and processed for antigen retrieval by heating in 10 mM citrate buffer (pH 6.0) at 90–100°C (FAK, VIMENTIN, FIBRONECTIN, E-CADHERIN) or digesting with 0.1% trypsin at 37°C (BrdU). Sections were incubated at 4°C overnight with FAK (3258, 1∶200, Cell Signaling Technology), VIMENTIN (550513, 1∶200, BD Pharmingen), FIBRONECTIN (610077, 1∶200, BD Transduction Laboratories), E-CADHERIN (610181, 1∶200, BD Transduction Laboratories) or BrdU (MAB0188, 1∶100, Maixin, Fuzhou, China ) antibody. Immunostaining was performed using UltraSensitive S-P Detection Kit (KIT-9720, Maixin, Fuzhou, China), and then color was developed by using a DAB kit (DAB-0031, Maixin, Fuzhou, China). Subsequently, sections were counterstained with hematoxylin. TUNEL assay was performed with an in situ cell death detection kit (Roche) according to the manufacturer’s instructions.Quantification of immunohistochemical stain intensity was performed as previously described [53].

### Luciferase Reporter Assay

Luciferase reporter assays were performed using the psiCHECK2-FAK-3′-UTR vector. Cells were grown to approximately 60% confluence in 24-well plates and cotransfected with psiCHECK2-FAK-3′-UTR (wild type or mutant) or psiCHECK2 empty vector plus miR-7 mimics or control mimics using Lipofectamine 2000 (Invitrogen). After 24 hours of incubation, Firefly and Renilla luciferase activities were evaluated using the Dual-Luciferase Reporter Assay system (Promega).

### Immunoblot

Cells were extracted in modified RIPA lysis buffer (150 mM NaCl, 50 mM Tris, pH 7.4, 1% NP-40, 0.25% Na-deoxycholate, 1 mM EDTA, protease inhibitor cocktail (Roche)). Proteins from total cell lysates were resolved by SDS-PAGE, transferred to the Nitrocellulose membrane (GE healthcare), blocked in 5% non-fat milk in PBS/Tween-20, and blotted with the antibodies for FAK (3258, 1∶1000, Cell Signaling Technology), phospho-FAK(Tyr^397^) (3283, 1∶1000, Cell Signaling Technology), VIMENTIN (550513, 1∶5000, BD Pharmingen), FIBRONECTIN (610077, 1∶5000, BD Transduction Laboratories), E-CADHERIN (610181, 1∶5000, BD Transduction Laboratories), N-CADHERIN (610920, 1∶2000, BD Transduction Laboratories), SNAIL (sc-28199, 1∶1000, SantaCruz Biotechnology) and GAPDH (M20028, 1∶5000, Abmart, Shanghai, China).

### Measuring Dendricity

Measuring cell dendricity was performed as previously described [54].

### Statistical Analysis

Data are presented as mean ± SD (standard deviation). Student’s *t* test (two tailed) was used to compare two groups, p<0.05 was considered significant.

## Supporting Information

Figure S1 **miR-7 target genes that predicted by all theree algorithms.** (A) Schematic illustration of target genes by three algorithms respectively. (B) 114 target genes of miR-7 that can be predicted by all three algorithms.
(TIF)

Figure S2 **Quantification of immunoblot bands intenstity and IHC DAB staining intensity.** (A) Quantification of immunoblot FAK and p-FAK bands intensity relative to GAPDH intensity in MDA-MB-435s cells are shown. (B) Quantification of immunoblot FAK and p-FAK band intensity relative to GAPDH intensity in MDA-MB-231 cells are shown. (C) Quantification of immunoblot FAK and p-FAK band intensity relative to GAPDH intensity in HBL-100 cells are shown. (D) Quantification of dendricty/inverse shape factor in MDA-MB-435s and MDA-MB-231 cells. (E) Quantification of relative DAB staining intensity in MDA-MB-435s derived tumor sections are shown.
(TIF)
