# Supplementary figures and images for: MicroRNA-7 Inhibits Epithelial-to-Mesenchymal Transition and Metastasis of Breast Cancer Cells via Targeting FAK Expression
Source: PLoS One. 2012 Aug 2;7(8):e41523. doi: 10.1371/journal.pone.0041523 (PMC3410899; doi:10.1371/journal.pone.0041523)

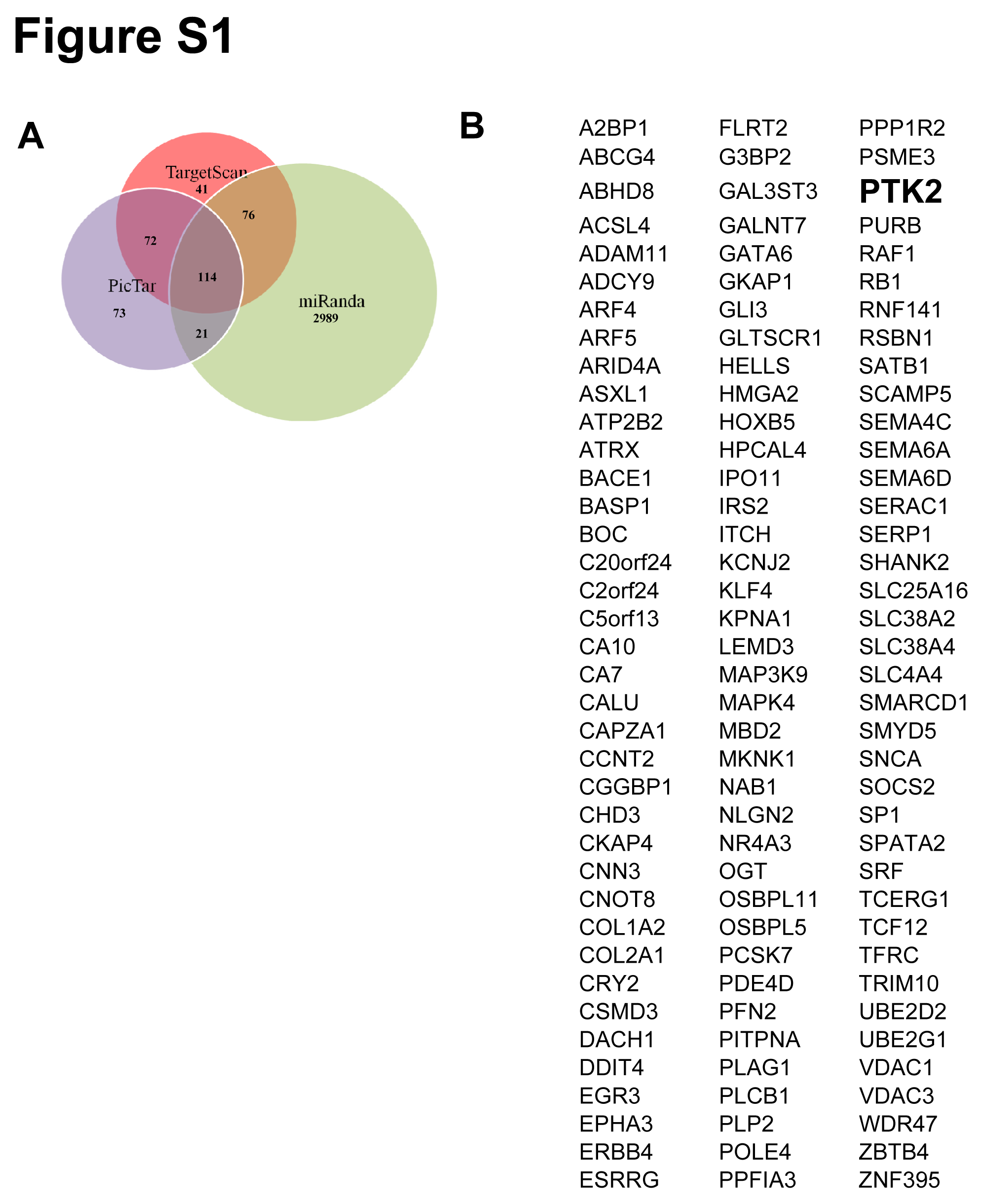

Supplement: Figure S1 — miR-7 target genes that predicted by all theree algorithms. (A) Schematic illustration of target genes by three algorithms respectively. (B) 114 target genes of miR-7 that can be predicted by all three algorithms. (TIF) [file pone.0041523.s001.tif]

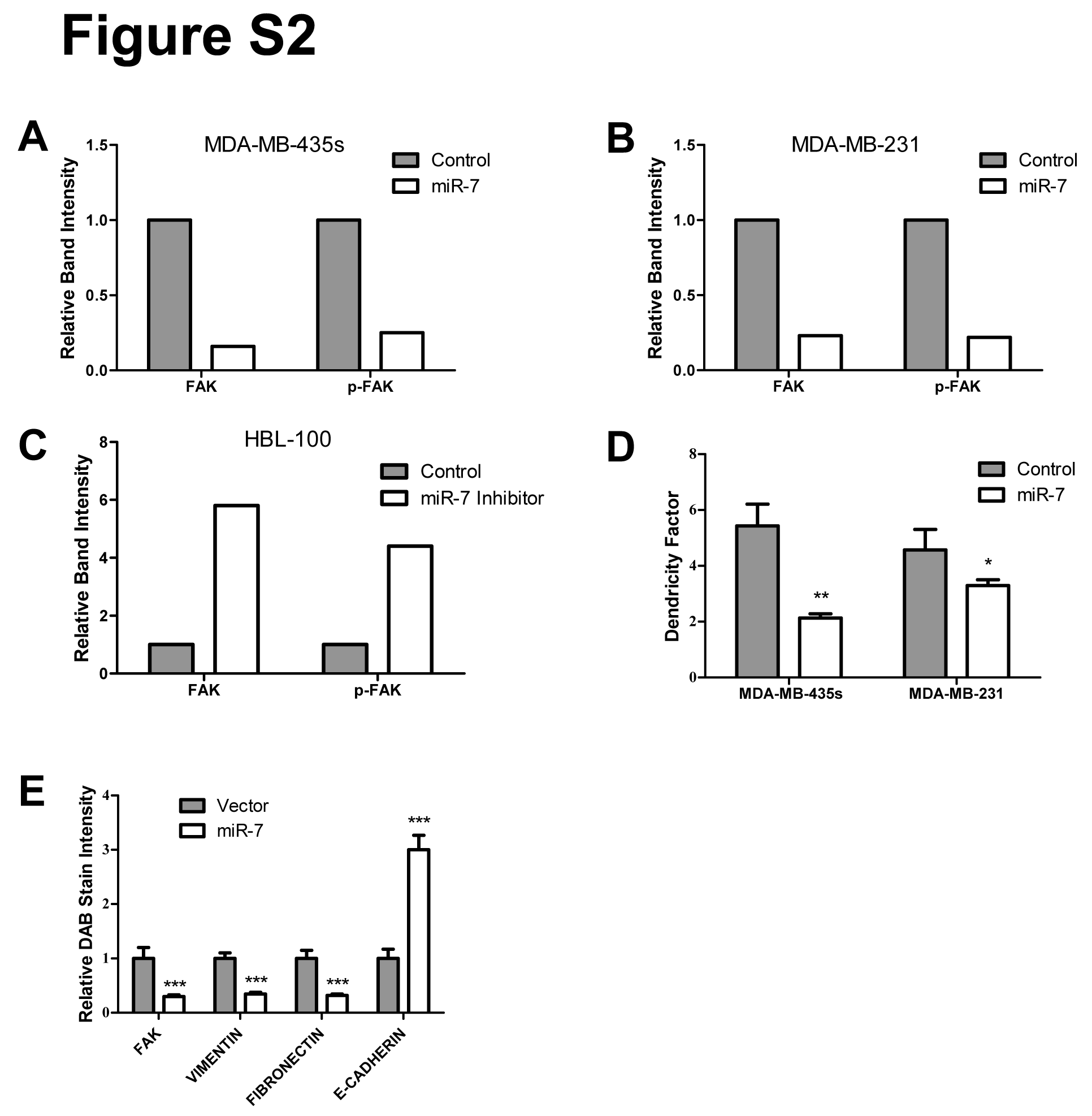

Supplement: Figure S2 — Quantification of immunoblot bands intenstity and IHC DAB staining intensity. (A) Quantification of immunoblot FAK and p-FAK bands intensity relative to GAPDH intensity in MDA-MB-435s cells are shown. (B) Quantification of immunoblot FAK and p-FAK band intensity relative to GAPDH intensity in MDA-MB-231 cells are shown. (C) Quantification of immunoblot FAK and p-FAK band intensity relative to GAPDH intensity in HBL-100 cells are shown. (D) Quantification of dendricty/inverse shape factor in MDA-MB-435s and MDA-MB-231 cells. (E) Quantification of relative DAB staining intensity in MDA-MB-435s derived tumor sections are shown. (TIF) [file pone.0041523.s002.tif]
